# Development of a Specialized Method for Simultaneous Quantification of Functional Intestinal Metabolites by GC/MS-Based Metabolomics

**DOI:** 10.1080/29933935.2024.2429408

**Published:** 2024-12-09

**Authors:** Kazuki Funahashi, Chol Gyu Lee, Kuniyo Sugitate, Noriko Kagata, Noriko Fukuda, Isaiah Song, Chiharu Ishii, Akiyoshi Hirayama, Shinji Fukuda

**Affiliations:** a Metagen Inc., Tsuruoka, Yamagata, Japan; b Agilent Technologies Japan, Ltd, Hachioji, Tokyo, Japan; c Institute for Advanced Biosciences, Keio University, Tsuruoka, Yamagata, Japan; d Gut Environmental Design Group, Kanagawa Institute of Industrial Science and Technology, Kawasaki, Kanagawa, Japan; e Transborder Medical Research Center, University of Tsukuba, Tsukuba, Ibaraki, Japan; f Laboratory for Regenerative Microbiology, Juntendo University Graduate School of Medicine, Bunkyo-ku, Tokyo, Japan

**Keywords:** Gut microbiome, intestinal metabolites, short-chain fatty acids, bile acids, GC/MS, TMS derivatization, volatility, metabolomics

## Abstract

Intestinal metabolites produced by gut microbes play a significant role in host health. Due to their different chemical structures, they are often analyzed using multiple analyzers and methods, such as gas chromatography/mass spectrometry (GC/MS) for SCFAs and liquid chromatography/mass spectrometry (LC/MS) for bile acids (BAs), amino acids (AAs), and sugars. In this study, we aimed to develop a specialized method for the simultaneous determination of important intestinal metabolites, specifically addressing the main issue of SCFA volatilization during the dry solidification process. We discovered that these compounds can all be measured in fecal samples by GC/MS after trimethylsilyl (TMS) derivatization despite the expected volatility of SCFAs. Validating the results using SCFA standards suggested that the fecal matrix exerts a stabilizing effect. This method enabled the simultaneous quantification of 65 metabolites. For further validation in a biological context, a mouse study showed that high-MAC and high-fat diets increased SCFAs and BAs in feces, respectively, and showed a negative correlation between *Alistipes* and sugars, all consistent with previous studies. As a result, we successfully developed a specialized simultaneous quantification method for SCFAs, BAs, AAs, AA derivatives, and sugars in fecal samples using GC/MS-based metabolomics in conjunction with a TMS derivatization pretreatment process.

## Introduction

Gut microbes metabolize undigested food components and produce a variety of metabolites, including short-chain fatty acids (SCFAs), bile acids (BAs), amino acids (AAs), and sugars. Their contribution to our health and implication in various diseases, such as infections, food allergies, cardiovascular disease, and intestinal inflammation, are well known.^1,2^

SCFAs identified in feces, such as formate, acetate, propionate, and butyrate, are interlinked with the host immune systems.^3^ Specifically, acetate is suggested to protect host epithelial cells, contributing to the prevention of infectious diseases *in vivo*.^4^ Propionate and butyrate have been shown to promote the epigenetic differentiation of regulatory T cells.^5^ Besides their association with the immune systems, SCFAs also contribute to diabetes prevention^6^ and endurance exercise performance.^7^ They are also linked to improvement of glucose tolerance,^8^ underscoring the intricate relationship between SCFAs in the gut and overall host health.

BAs, amphipathic digestive compounds derived from the host, are secreted into the duodenum from the bile duct in response to meal intake. Although 95% of these acids are reabsorbed in the small intestine, a portion remains within the intestinal tract, where it is metabolized by gut microbes. The resulting BAs induce germination of spore-forming bacteria,^9^ regulate the expression of pathogenic bacterial genes,^10^ ameliorate influenza virus and SARS-CoV-2 infections,^11^ and are thus instrumental in maintaining gut homeostasis.

Recent studies of the intestinal metabolome, extending beyond SCFAs and BAs, have further illuminated its connection to host health. For instance, levels of proline, an amino acid derived from gut microbes, are elevated in individuals suffering from depression.^12^ Indole-3-acetic acid, a metabolite derived from tryptophan, an essential amino acid, has also been reported to influence chemotherapy efficacy in pancreatic cancer.^13^ Such observations illustrate the systemic influence of gut-derived AAs. Similarly, monosaccharide-utilizing bacteria correlated with insulin resistance exist in the gut, linking the presence or absence of certain sugars directly to host blood glucose levels.^14^ These findings demonstrated that SCFAs and BAs, as well as AAs and sugars, are metabolites that play functional roles in the gut.

Although the value of studying the intestinal metabolome is clear, no analytical method has been reported that can simultaneously quantify SCFAs, BAs, AAs, and sugars in fecal samples. While a ultra-performance liquid chromatography-quadrupole time-of-flight mass spectrometry (UPLC-Q/TOF-MS) method specific for the intestinal metabolome can be found in the literature, this method is not able to measure BAs, which are deeply implicated in intestinal health.^15^ This limitation arises from the distinct properties of each metabolic compound, such as polarity, structure, and molecular weight. Indeed, several studies have successfully quantified SCFAs and BAs in human feces, but separate measurement systems were necessary to achieve this.^11,16,17^

Two major pretreatment methods exist for gas chromatography/mass spectrometry (GC/MS): metabolite extraction from fecal samples and post-extraction derivatization. For water-soluble and nonvolatile AAs and sugars, a known method involves extracting the water-soluble fraction via liquid-liquid separation in a water/methanol/chloroform mixture, followed by centrifugal drying/lyophilization and a two-step derivatization process consisting of oximization and trimethylsilylation (TMS) before quantification by GC/MS.^18^ In contrast, BAs are extracted using methanol as the solvent, followed by centrifugal solidification/lyophilization, a two-step derivatization through esterification and TMS, and subsequent quantification by GC/MS.^19–21^ It has been demonstrated that a combination of water/methanol solvent followed by drying and subsequent oximization and TMS derivatization enable the quantification of metabolites with diverse characteristics, such as BAs, AAs, and sugars, using the same pretreatment.^22^ However, while this approach could detect C5 and C6 SCFAs, C2, C3, and C4 SCFAs (acetic, propionic, and butyric acids) could not be detected. Due to the volatilization of these SCFAs during the pretreatment processes, such as dry solidification, quantifying these vital metabolites in studying the gut environment is challenging.^23^ As an alternative, diethyl ether extraction under acidified conditions using hydrochloric acid and subsequent derivatization with tert-butyldimethylsilyl has been proposed.^5^ However, Ueyama et al. (2020)^24^ showed that volatile SCFAs, such as acetic acid and butyric acid, can be stably preserved when treated and processed with feces without prior purification. Their volatilization was significantly reduced even after drying treatment. This study suggests a potential solution to the previously insurmountable issue of SCFA volatilization post-lyophilization, potentially enabling the simultaneous quantification of various metabolites, including SCFAs. Based on these studies, we hypothesized that SCFAs, BAs, AAs, and sugars could undergo the same pretreatment process and be simultaneously quantified by GC/MS after TMS derivatization.

We first evaluated the quantification of SCFAs during the dry solidification process prior to TMS derivatization. To ensure accurate quantification, we applied the standard addition method approach. This technique is especially useful when the complexity of the samples may interfere with the measurement of specific components. By adding a known quantity of standards to fecal samples, we could create calibration curves for our analysis. The effectiveness and reliability of this method were verified through tests on mouse feces, to confirm its suitability for analyzing biological samples. The goal of this study is to develop a specialized method for simultaneous quantification of functional intestinal metabolites, such as SCFAs, BAs, AAs, and sugars, in fecal samples using a comprehensive pretreatment and processing method.

## Materials & methods

### Chemicals, reagents, instruments, and quality control fecal samples

Standards were purchased from CHEM SERVICE, Kanto Chemical (Tokyo, Japan), TCI (Tokyo, Japan), SIGMA-Aldrich (STL, USA), ICN (CA, USA), Fluka (NC, USA), FUJIFILM Wako (Osaka, Japan), Cayman Chemical (MI, USA), and others. Ribitol used as an internal standard was purchased from Wako. Myristic acid was from the Fiehn GC/MS Metabolomics standards kit (Agilent 400,505). N-Methyl-N-(trimethylsilyl)trifluoroacetamide (MSTFA) and methoxyamine were purchased from SIGMA-Aldrich. Quality control (QC) fecal samples were prepared by mixing 10 freeze-dried human fecal samples.

### Evaluation of SCFA volatility with or without fecal co-treatment

To verify whether SCFA samples volatilize during the extraction and derivatization procedures, we tested the effects of fecal co-treatment on measurement of SCFAs. Acetic acid, propionic acid, and butyric acid were used for the validation. The analysis compared the relative peak areas of 2,500 µM standard solutions of each SCFA with and without the addition of QC feces, and the relative area of the samples added with QC feces was calculated by subtracting the relative area of the QC feces alone. Standard solutions of each SCFA were analyzed in two forms: as salts and in their free states. The extraction and derivatization procedures are described below.

### Extraction and derivatization of metabolites from fecal samples

**Figure 1.**
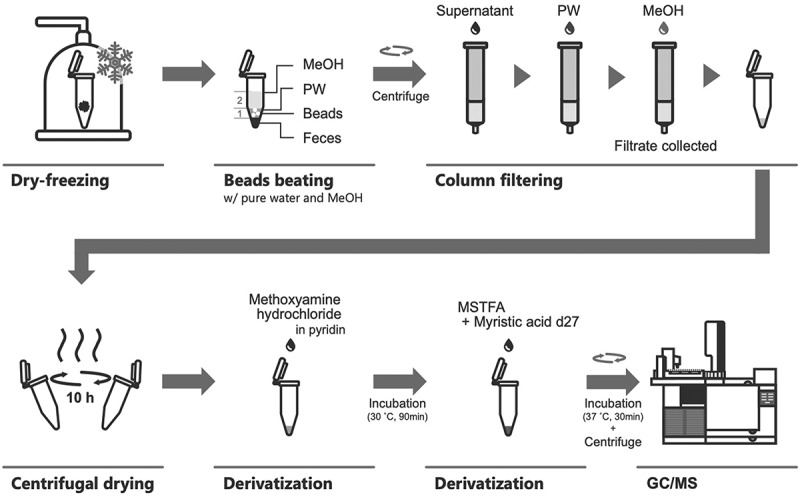
Overview of sample handling protocol for intestinal metabolome analysis in this study.

The experimental procedure is shown in Figure 1 Fecal samples were freeze-dried for at least 24 hours using a VD-800 R freeze-dryer (TAITEC, Japan). They were then subjected to vigorous shaking with 3.0 mm zirconia beads using a Shake Master (Biomedical Science, Japan) to rupture the cell membrane (1,500 rpm., 10 minutes). After disruption, 10 mg of the samples were weighed, and 0.1 mm zirconia/silica beads were added. Then, 600 μL of a water:methanol (1:2) solution containing ribitol (167 μM) as an internal standard was added. The mixture was then shaken again for 10 minutes and centrifuged (20°C, 4,600 × g, 10 min). Then, 300 μL of supernatant was transferred to Bond Elut C18 (Agilent 12,102,058) and dispensed into a 2 mL tube. The hydrophilic compound was extracted with 500 μl of water, and the hydrophobic compound with 600 μl of methanol. The mixture was dried using a centrifugal concentrator under reduced pressure (40°C, 10 hours). After drying, 20 μl of methoxyamine hydrochloride (20 mg/mL, pyridine solution) was added and the mixture was incubated under 30°C for 90 min. Then, 70 μl of N-Methyl-N-trimethylsilyl trifluoroacetamide (MSTFA) and 10 μl of myristic acid-d27, serving as an internal standard for retention time locking, were added. The TMS derivatization was performed at 37°C for 30 minutes.^18^

### GC/MS analysis

The GC/MS analysis was performed using an Agilent 5977B GC/MS with the split-splitless inlet, equipped with electron impact (EI) ion source. A DB-5MSUI fused-silica capillary column (30 m, 0.25 mm, 0.5 μm, 122-5536UI) was utilized to separate the derivatives. The column thickness generally used in metabolomics is 0.25 μm, but 0.5 μm was employed because the retention time of SCFAs is buried in the peak of the TMS derivatization reagent when 0.25 μm is used. Hydrogen was used as a carrier gas at a constant flow rate of 1.5 mL/min through the column. One microliter of the sample was injected in the split mode at a ratio of 1:50. The solvent delay time was set to 1.8 min. The initial oven temperature was held at 30°C for 2.76 min, ramped to 100°C at a rate of 9 °C/min, to 180°C at a rate of 18 °C/min, to 310°C at a rate of 2 °C/min, to 325°C at a rate of 18 °C/min, and finally held at 325°C for 8.27 min. The temperatures of the injector, transfer line, and EI ion source were set to 250°C, 300°C, and 250°C, respectively. The electron energy was 70 eV, and mass data was collected in a sim/scan mode (m/z 50–700).

### Validation of compound measurement methods

To evaluate the quantitative performance of our method, we assessed the linearity, linearity range, recovery rate, and precision. The compounds used to validate these assessment items are listed in Supplementary Table S1. Verified compounds include SCFAs, BAs, AAs, sugars, as well as vitamins and indole compounds.

Calibration curves for each metabolite were created by plotting the peak area ratio of the analyte to the internal standard (ribitol) against the analyte concentrations. The calibration curves were prepared using a QC fecal sample to account for volatilization potential of SCFAs. We employed 10% QC samples, prepared by diluting QC samples to one-tenth of their original concentrations. This dilution strategy enables a precise examination of SCFA’s behavior at lower concentrations, providing insights into the method’s sensitivity and specificity across the analyte concentration range (Table 1). Linearity was assessed by the coefficient of determination (R^2^) for the linear regression between concentration and relative peak area of each metabolite. Good linearity was defined as R^2^ > 0.95. To prove the reproducibility of the intra-assay response to each analyte, the intra-assay precision was determined with three technical fecal sample replicates at three concentration levels.

**Table 1.** The study design for evaluating sample handling protocols in fecal metabolome analysis.

Experiment	Fecal samples used	Objective
Volatility	100% QC human feces	To verify whether mixing SCFA standards in feces would reduce volatilization
Linearity	10% QC human feces	To verify the linearity of the calibration curve
Recovery	10% QC human feces (calibration curve) 100% single feces (test sample)	To verify recovery rates in an actual fecal sample
Repeatability	100% single feces	To verify stability of repeated measurements on an actual fecal sample
Mouse	10% QC mouse feces (calibration curve) 100% single feces (test samples)	To verify if the method is sufficient to evaluate differences in the amount of metabolites in feces

To evaluate instrument precision and recovery, a standard mixture of metabolites was spiked into a 100% single feces sample to mimic the composition of actual fecal samples without dilution. This approach ensures that our analysis reflects conditions as close to natural fecal samples as possible. For evaluating precision, 10 repeated measurements were conducted once, and five repeated measurements were performed twice on separate days. The precision of metabolites was assessed in terms of intra-day and inter-day variability and expressed as relative standard deviation (RSD) percentage. The precision level was deemed acceptable if less than 15%.^25^

The recovery was determined by comparing the concentration of metabolites in fecal samples spiked with mixed standard solutions before extraction. This was further verified by adding specific concentrations of the samples to 600 µL of a water:methanol (1:2) solution and analyzing as described extraction method. The concentrations of compounds added are listed in Supplementary Tables S2 and S3. The recovery rate was calculated as (pre-extraction concentration/post-extraction concentration) × 100 at each spiking level. The acceptable recovery rate ranged from 70 to 130%.^25^

Compounds that met all of these criteria were considered to have passed quantitative validation.

In addition, the limit of detection (LOD), limit of quantification (LOQ), and matrix effect were also evaluated.

The signal-to-noise (S/N) ratio for determining the limit of detection (LOD) and the limit of quantification (LOQ) was set to a minimum value of 3 and 10, respectively. LOD and LOQ were calculated by the following method:

LOD = 3 × concentration of standard/(S/N of Standard with 10% QC fecal sample – S/N of 10% QC fecal sample)

LOQ = 10 × concentration of standard/(S/N of Standard with 10% QC fecal sample – S/N of 10% QC fecal sample)

The matrix effect was calculated according to the following formula by comparing the relative signal intensity between the 10% QC sample and the actual fecal sample when a standard is added to each at the same concentration:

Matrix effect (%) = (relative area of actual fecal sample with standard – relative area of actual fecal sample)/(relative area of 10% QC fecal sample with standard – relative area of 10% QC fecal sample)

For analytes producing multiple peaks, the peaks to be selected were chosen based on three criteria: linearity of the calibration curve, strength of the signal intensity, and absence of nearby conflicting peaks. In particular, the use of the m/z 73 of the derivatization reagent was avoided.

### Mouse samples and animal treatment

Male C57BL/6J mice, 6 weeks old (*n* = 4), were obtained from CLEA Japan, Inc. (Tokyo, Japan) and were fed a mixed diet consisting of AIN-93 G (EP Trading, Tokyo, Japan), CE-2 (CLEA Japan, Tokyo, Japan) and D12492 (EP Trading, Tokyo, Japan). After acclimation, AIN-93 G (Control), D12492 (High Fat), and CE-2 (High microbiota-accessivle carbondydrates (MAC)) were each fed every week (Figure 4a). At the end of each week, mouse fecal samples were collected and stored at − 80 ◦C for further analysis.

Processed and having metabolites extracted similarly to human fecal samples, calibration curves for the concentration and relative area of each metabolite in the mouse study were created using a baseline of 10% mouse QC feces.

### Microbiome analysis from mouse fecal samples

The extraction and measurement of fecal microbial DNA were performed as previously described.^26^ Briefly, the fecal samples were initially lyophilized and shaken vigorously using a Shake Master. Samples were then suspended in DNA extraction buffer containing 400 μL of a 1% w/v SDS/TE (10 mm Tris-HCl, 1 mm EDTA; pH 8.0) solution, and fecal samples in the buffer were further shaken with 0.1 mm zirconia/silica beads using a Shake Master (1,500 rpm, 5 min). After centrifugation (20°C 17,800 × g, 10 min), bacterial DNA was extracted using an automated DNA extraction machine according to the instruction manual (GENE PREP STAR PI-480). After DNA extraction, the V1–V2 variable region of the 16S rRNA gene was amplified using the bacterial universal primers 27F-mod (5′-AGRGT TTGATYMTGGCTCAG-3′) and 338 R (5′-TGCTGCCTCCC GTAGGAGT-3′) with Tks Gflex DNA Polymerase (Takara Bio Inc., Japan).^27^ Amplicon DNA was sequenced using MiSeq (Illumina, USA), according to the manufacturer’s protocol.

### Bioinformatics analysis

For 16S rRNA gene-based microbiome analysis, QIIME2 (version 2019.10) was used.^28^ Primer bases were trimmed using cutadapt (option: –p-discard-untrimmed).^29^ Sequence data were processed using the DADA2 pipeline for quality filtering and denoising (options: –p-trunc-len-f 230 –p-trunc-len-r 130).^30^ Contamination by the human genes was checked by mapping the filtered output sequences, and no contamination was found. The filtered output sequences were assigned to taxa using the “qiime feature-classifier classify-sklearn” command with the default parameters.^31^ Silva SSU Ref Nr 99 (version 132) was used as the reference database for taxonomic assignment. Alpha and beta diversities were calculated using “qiime phylogeny align-to-tree-mafft-fasttree” and “qiime diversity core-metrics-phylogenetic” commands with the sampling depth set to the lowest read numbers.

### Statistical analysis

All statistical analyses were performed using Python scripts (version 3.7.6). For beta-diversity analysis, microbiome unweighted/weighted UniFrac distance and metabolome spearman correlation distance were used. Distance matrices were visualized via principal coordinate analysis (PCoA) analysis. Each metabolite value was standardized by centering to a mean of 0 and dividing by the standard deviation (z-score) of each metabolite. Z-score was obtained by normalization among all samples. Spearman rank correlation coefficient was used to validate the associations between gut bacteria and metabolites (scipy version 1.5.2). Visualization was performed using Cytoscape 3.10.2 © software based on Spearman correlations between gut bacteria and intestinal metabolites.

## Results

### Verification of SCFA volatility during the preprocessing step

**Figure 2.**
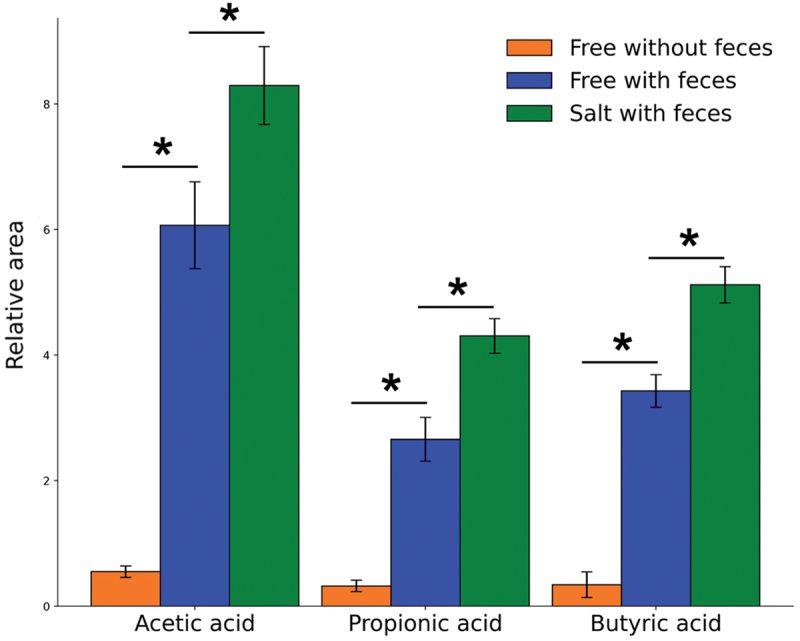
Volatility of the SCFAs acetic acid, propionic acid, and butyric acid in the pretreatment process. Comparison of relative SCFA standard quantities as measured in processed samples between standard in free form (orange), standard in free form co-treated with feces (blue), and standard in salt form co-treated with feces (green). As this experiment would be used as a theoretical baseline for constructing a future calibration curve, absolute concentrations could not be determined as of this step. Relative areas are based on initial SCFA standard concentrations of 2,500 μM, determined based on naturally quantified amounts in human feces.

First, we tested for the potential loss of SCFA in the pretreatment step, especially in the evaporation step after solid-phase extraction (SPE). The relative peak areas of pure acetic acid, propionic acid, and butyric acid were significantly diminished in comparison to those co-treated with feces at a free standard concentration of 2,500 μM (Figure 2). Specifically, the relative peak areas of acetic acid, propionic acid, and butyric acid were reduced by factors of 11.0, 8.2, and 10.0, respectively. These results indicate that the addition of feces before pretreatment suppressed volatilization. The relative peak areas of the SCFA salt standards co-treated with feces were significantly higher than those of free SCFA standards, with increases of 1.4 times for acetic acid, 1.6 times for propionic acid, and 1.5 times for butyric acid. This result suggests that SCFAs in salt form further resist volatilization (Figure 2).

### Quantitative validation of SCFAs

**Figure 3.**
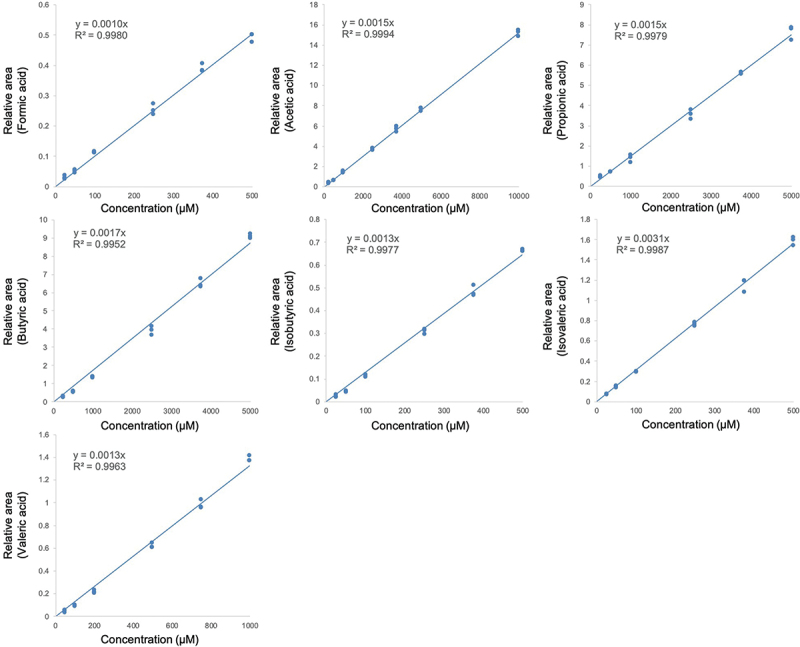
Calibration curves of each SCFA calculated using baseline QC fecal measurement ranges as a reference. Analyzed standards were SCFA salts co-treated with fecal samples during pretreatment.

The R^2^ of the calibration curve using 10% QC feces sample exceeded 0.990 for all compounds within the test ranges (Figure 3, Supplementary Table S2). The linear ranges were 250–10,000 μM for acetic acid, 250–5,000 μM for propionic acid and butyric acid, 50–1,000 μM for valeric acid, and 25–500 μM for formic acid, isobutyric acid, and isovaleric acid (Supplementary Table S2), determined according to concentration ranges seen in actual fecal samples.

The RSDs for the intra-day precision and inter-day precision were 10.0% and 13.3% for formic acid, 3.9% and 5.0% for acetic acid, 0.8% and 4.1% for propionic acid, 3.6% and 3.9% for isobutyric acid, 1.9% and 2.4% for butyric acid, 4.0% and 4.8% for isovaleric acid, and 4.0% and 5.2% for valeric acid (Supplementary Table S2).

The recovery rates were 82.3% for formic acid, 98.1% for acetic acid, 96.9% for propionic acid, 110.1% for isobutyric acid, 93.6% for butyric acid, 97.6% for isovaleric acid, and 88.5% for valeric acid (Supplementary Table S2). Overall, these results demonstrate an acceptable level of linearity, inter-day and intra-day precision, and high rates of SCFA recovery. All metrics support the validity of our method for SCFA quantification.

### Quantitative validation of BAs, AAs, sugars, and other compounds

The same validation methods were similarly applied for other compounds, such as BAs, AAs, and sugars (Supplementary Table S3). Galactose and talose could not be separated, so quantitative validation was performed for the combined concentration of these two compounds. The R2 of the calibration curve using the 10% QC feces are between 0.959–0.998 for BAs, 0.961–0.996 for AAs, 0.975–0.993 for sugars, and 0.957–0.997 for other compounds within the test range (Supplementary Table S3). The linear ranges were 2.5–1,000 µM for BAs, 25–1,000 µM for AAs, 25–1,000 µM for sugars, and between 2.5–1,000 µM for other compounds. As these ranges include all compounds within each group, the linear range for each specific compound can be found in Supplementary Table S3.

The RSDs for intra-day precision and inter-day precision were 4.1–11.4% and 6.7–13.3% for BAs, 2.2–11.4% and 4.8–12.8% for AAs, 2.3–12.5% and 6.9–13.9% for sugars, and 1.9–12.4% and 4.0–14.8% for other compounds (Supplementary Table S3). These results suggested that the method exhibited acceptable precision for these metabolites.

The recovery rates were 91.2–118.2% for BAs, 77.3–114.2% for AAs, 89.7–122.4% for sugars, and 72.4–114.9% for other compounds (Supplementary Table S3).

Quantitative validation was performed for 87 compounds other than SCFAs (Supplementary Table S1) and the compounds that passed quantitative validation, consisting of 10 bile acids, 14 amino acids, 12 sugars, 5 vitamins, and 17 other compounds (Supplementary Table S3). The retention time, m/z, and chromatographic information for these compounds are shown in Supplementary Figure S1.

### Comparison of mouse fecal metabolites with dietary variation

Having confirmed the validity of our sample processing and quantification method, we tested the method in a biological model alongside fecal microbiome sequencing results to simulate a plausible application in mouse gut studies. Since previous studies have reported increased SCFAs with high-MAC diets and increased BAs with high-fat diets in mouse studies, we tested whether our results would be consistent with these findings.^5,32^ We also measured correlations between metabolite concentrations and the gut microbiome to compare them to trends reported in the literature and further validate our metabolite quantification method. In particular, *Alistipes* and *Bacteroides* are monosaccharide-utilizing bacteria that have been reported to be negatively correlated with intestinal monosaccharide levels.^14^ We focused on these two genera in our experiment to compare their correlations with intestinal metabolites to those found in the literature.

**Figure 4.**
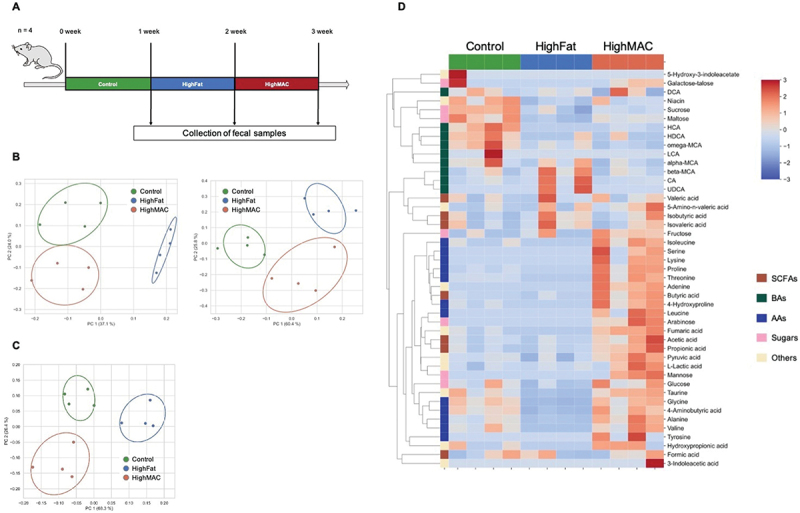
Mouse test to demonstrate the applicability of the proposed method to biological samples. A) experimental overview of mouse conditions. Individuals (*n*=4) were fed a combination of control (AIN-93G), high-fat (D12492), and high-MAC (CE-2) diets for one week each. Fecal samples were collected after each week, and metabolome analysis was performed using the method developed in this study. B) PCoA based on unweighted (left) and weighted (right) UniFrac distances of fecal microbiome compositions and C) Spearman correlation of fecal metabolome data. D) hierarchical clustering analysis heat map of intestinal metabolites (Spearman correlation distance, average-linkage method), represented by z-scores. Z-score was obtained by normalizing all samples of a given metabolite.

**Figure 5.**
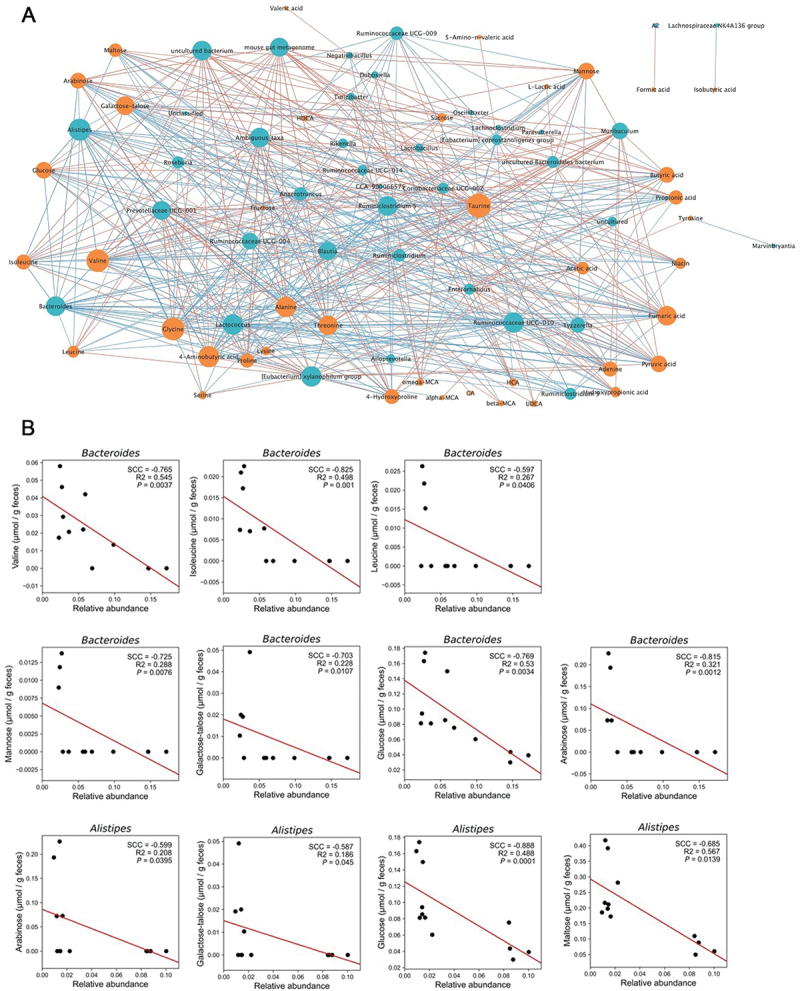
Spearman correlation between gut bacteria and intestinal metabolites in mice. A) Cytoscape network analysis of Spearman correlation coefficient (SCC) between gut bacteria and intestinal metabolites. Relative abundance ratios of gut bacteria that were greater than 0.001 and *p* values less than 0.05 were drawn. B) correlation analysis between *Alistipes/Bacteroides* and intestinal metabolites in mouse feces. Plots are arranged by metabolite type: BCAAs (top row), and sugars (middle, bottom rows).

In this experiment, we quantified the fecal metabolome in mice prescribed a three-week dietary regimen, with the diet changing every week between a control diet, high-fat diet, and high-MAC diet (Figure 4A). A total of 45 of the 65 validated compounds were successfully quantified in mouse feces in at least one of the samples. The composition of intestinal metabolites also changed with diet, as expected (Figure 4C). The cluster map showed that increased levels of SCFAs and AAs were characteristic of the high-MAC diet group (Figure 4D). Specific bile acids such as cholic acid, β-muricholic acid, and ursodeoxycholic acid were elevated in the high-fat diet group (Figure 4D). Other bile acids were more prevalent in the control diet group, indicating a diverse response of intestinal metabolites to different dietary regimens. We analyzed the correlations between intestinal metabolites and gut bacteria, and showed those with significant correlations (Figure 5A). Among them, significant negative correlations (p-value <0.05) were observed between *Bacteroides* and branched-chain amino acids (valine, leucine, isoleucine) (Figure 5B). Similarly, significant negative correlations were observed between *Alistipes*, *Bacteroides* and monosaccharides (arabinose, glucose, galactose, talose) (Figure 5B). The gut microbiota composition also distinct between each dietary group (Figure 4B).

These factors make it important to quantify intestinal functional metabolites such as SCFAs, BAs, AAs, and sugars in gut environment studies. The effectiveness and reliability of the pretreatment method were verified through tests on mouse feces.

## Discussion

This study investigated the feasibility of quantifying SCFAs and other metabolites in fecal samples using a comprehensive pretreatment method for GC/MS analysis. A primary challenge identified was the volatilization of SCFAs during the drying and solidification process.^23^ Our study showed that the volatilization rate of SCFAs co-treated with feces was significantly lower than that of SCFAs in isolation, and their recovery rates from fecal mixtures exceeded 80%. These results suggest that SCFAs in feces are less susceptible to volatilization during the drying after SPE than pure SCFA solutions, presumably due to the formation of fatty acid salts within the fecal matrix. Fecal samples typically contain high levels of potassium and sodium^33^ that can react with free SCFAs to form stable fatty acid salts such as sodium acetate, with a higher boiling point (approximately 400 °C) compared to acetic acid (118°C). Although the exact proportion of SCFAs converted into salts in feces is unknown, the acceptably high recovery rate (Supplementary Table S2), robust calibration curves (Figure 3), and broad SCFA coverage in human feces^34^ indicate that a majority of the SCFAs in feces likely exist as salts. The successful development of a simultaneous measurement system for SCFAs, BAs, sugars, and AAs and their derivatives in this study demonstrates the efficacy of the pretreatment method.

Biological tests conducted in mice have verified the applicability of this method for gut environment research. Previous studies showed that a high-MAC diet increases SCFAs such as acetic acid, butyric acid, and propionic acid, which are by-products of fiber digestion.^5^ Conversely, a high-fat diet increases primary BAs like cholic acid and β-muricholic acid,^32^ supporting their role in fat digestion and absorption.^32^ Interestingly, contrary to findings in previous studies, other bile acids were found to be characteristically elevated in the control diet group. This may be because the cellulose in the control adsorbed bile acids, preventing them from reentering circulation and to instead be released in feces.^35^ Additionally, negative correlations were observed between the relative abundance of *Bacteroides* and the concentrations of branched-chain amino acids including valine, leucine, and isoleucine, aligning with the results of a previous study.^36^ For sugars, significant negative correlations were observed between *Alistipes* and monosaccharides, such as arabinose, glucose, galactose & talose, also consistent with a previous study.^14^ These results indicate that the method is sufficiently applicable to actual fecal samples, allowing for simultaneous quantification of SCFAs, BAs, AAs, and sugars – key compounds in gut environmental studies.

There are two limitations of this method. First, this method does not distinguish between galactose and talose, resulting in a combined analysis of these sugars. For more detailed analysis of sugars, using an alternative analytical instrument like LC/MS/MS would be preferable. However, it is worth noting that talose is a rare sugar, so most of the combined galactose and talose value is likely attributable to galactose.^37^ Second, it cannot measure conjugated BAs due to their large molecular weight, which precludes detection by GC/MS. To include these BAs, hydrolysis would need to be added as a pretreatment. However, it is worth noting that fecal bile acids are rarely found to be conjugated and hydrolysis treatment may not be necessary.^38^

Consequently, these limitations may affect the reproducibility of the method compared to prior studies where LC/MS/MS was used to distinguish between galactose and talose and separately measure conjugated BAs.

While this method has been shown to be able to measure with high accuracy, other measurement methods such as LC/MS/MS are admittedly more sensitive for LOD and LOQ of SCFAs.^39^ The quantitative range is narrower than that of targeted analysis methods. With that said, in the case of SCFAs, the concentration ranges observed in human feces are well-within the quantitative ranges established in our method.^40^ Therefore, although other methods are more suitable for specialized applications, our method is both convenient and sufficient for a comprehensive analysis of the key metabolites in gut microbiome research. It is also worth noting that this method is much more rapid due to less processing of samples and also requires less sample material, which addresses a common issue in mouse fecal analytical studies. The GC/MS equipment is also more widely available, allowing this method to be more accessible to researchers as well as being more cost-efficient. Despite certain limitations, this method is a valuable tool for comprehensive studies of intestinal environments.

In conclusion, the study successfully established a specialized method for simultaneous determination of functional intestinal metabolites such as SCFAs, BAs, AAs, and sugars. Notably, despite the volatility of SCFAs, this study showed that SCFAs in feces do not volatilize to the point of loss of quantitation even after drying and solidification. This method can also quantify additional compounds like AAs and sugars, offering a comprehensive tool for gut environment analysis. This method can be used as a first step in intestinal metabolome analysis due to its ability to measure functional metabolites in the gut in a relatively comprehensive manner. Although there are some limitations, we expect gut environmental research to progress in the future using the method.

## Supplementary Material

SupplementaryFigure1I.png

SupplementaryFigure1A.png

SupplementaryFigure1H.png

SupplementaryFigure1J.png

SupplementaryFigure1G.png

SupplementaryFigure1D.png

SupplementaryFigure1E.png

SupplementaryFigure1C.png

SupplementaryTable.xlsx

SupplementaryFigure1F.png

SupplementaryFigure1B.png

## Data Availability

The microbiome data have been deposited with links to BioProject accession number PRJDB18118 in the DDBJ BioProject database. Data used for analysis and the in-house scripts for performing bioinformatics analysis in this work can be found on GitHub at https://github.com/metagen/article_GC-MSmetabolomics.

## Abbreviations

SCFA: Short-chain fatty acid
BA: Bile acid
AA: Amino acid
GC/MS: Gas Chromatography/Mass Spectrometry
TMS: Trimethylsilylation
