# Supplementary figures and images for: Development of a Specialized Method for Simultaneous Quantification of Functional Intestinal Metabolites by GC/MS-Based Metabolomics
Source: Gut Microbes Rep. 2024 Dec 9;1(1):2429408. doi: 10.1080/29933935.2024.2429408 (PMC12940120; doi:10.1080/29933935.2024.2429408)

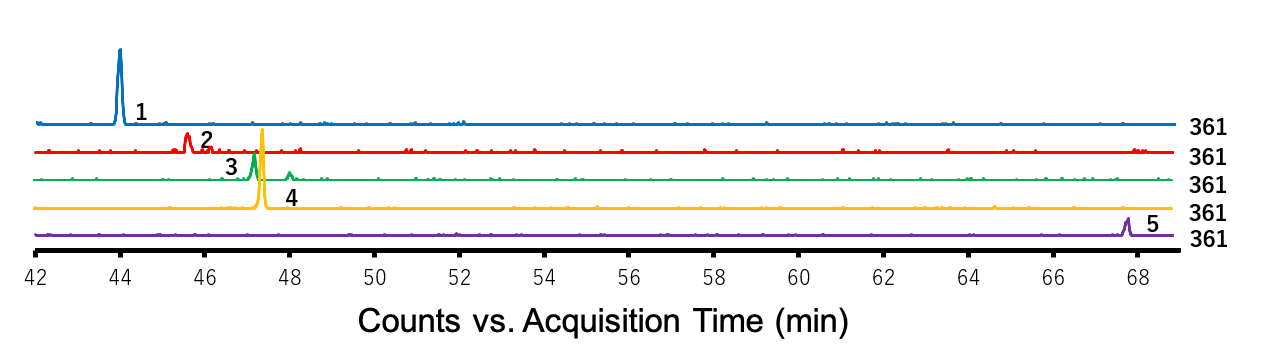

Supplement: SupplementaryFigure1I.png [file KGMR_A_2429408_SM5721.png]

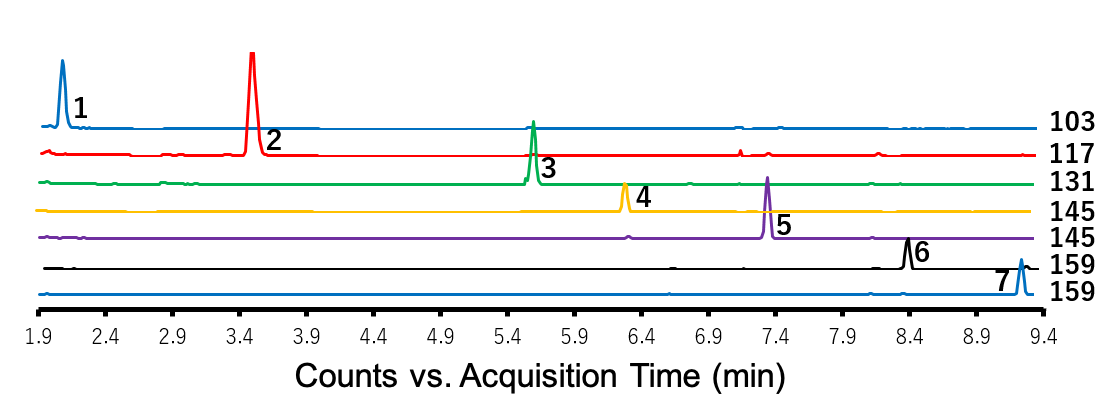

Supplement: SupplementaryFigure1A.png [file KGMR_A_2429408_SM5720.png]

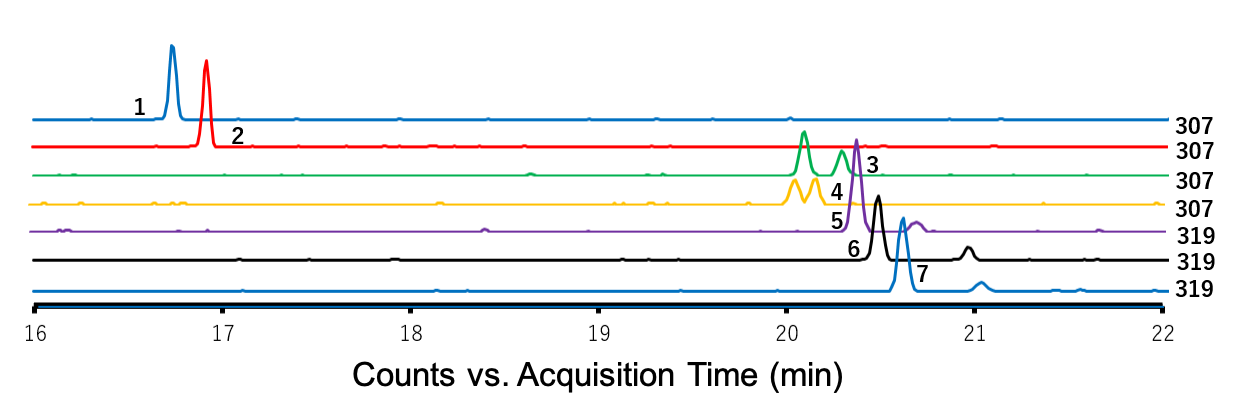

Supplement: SupplementaryFigure1H.png [file KGMR_A_2429408_SM5719.png]

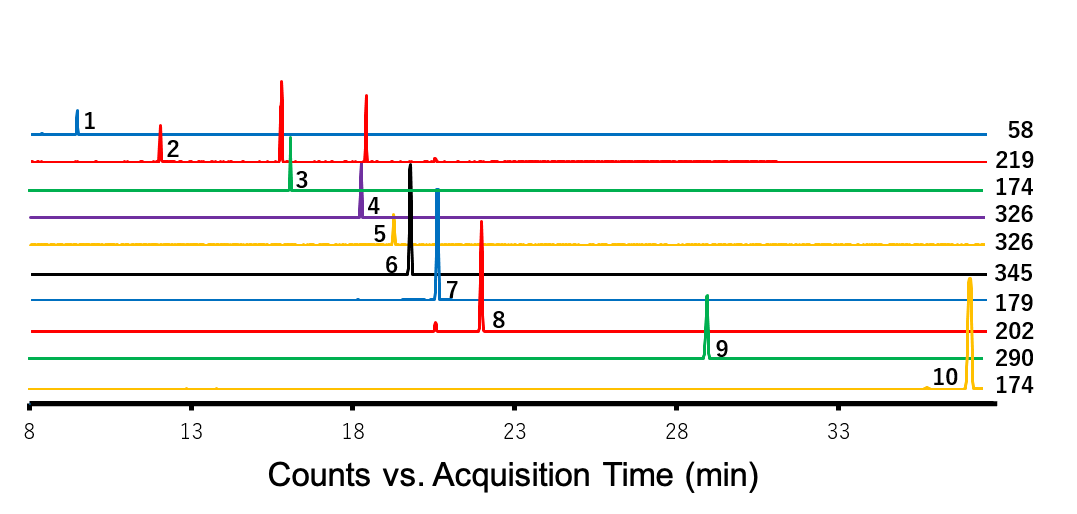

Supplement: SupplementaryFigure1J.png [file KGMR_A_2429408_SM5718.png]

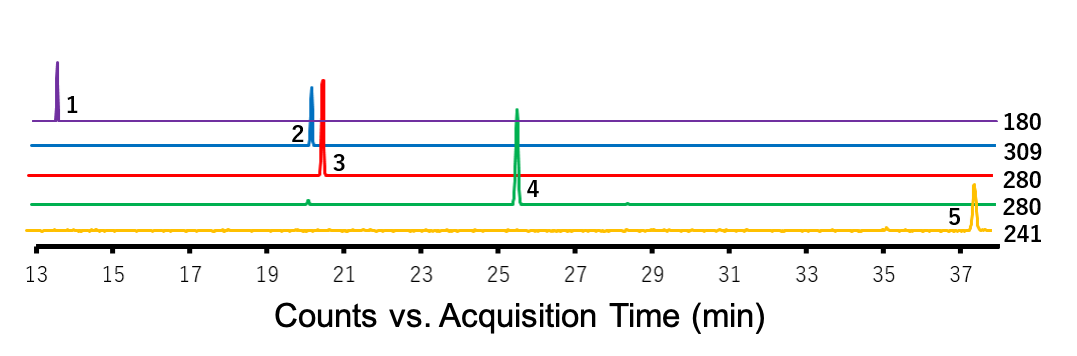

Supplement: SupplementaryFigure1G.png [file KGMR_A_2429408_SM5717.png]

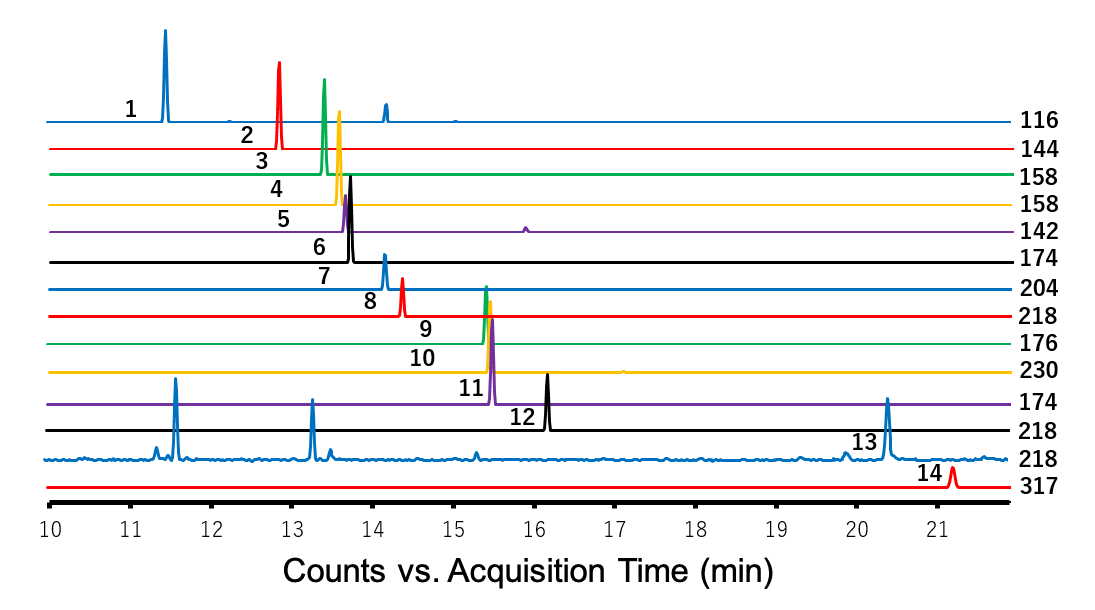

Supplement: SupplementaryFigure1D.png [file KGMR_A_2429408_SM5716.png]

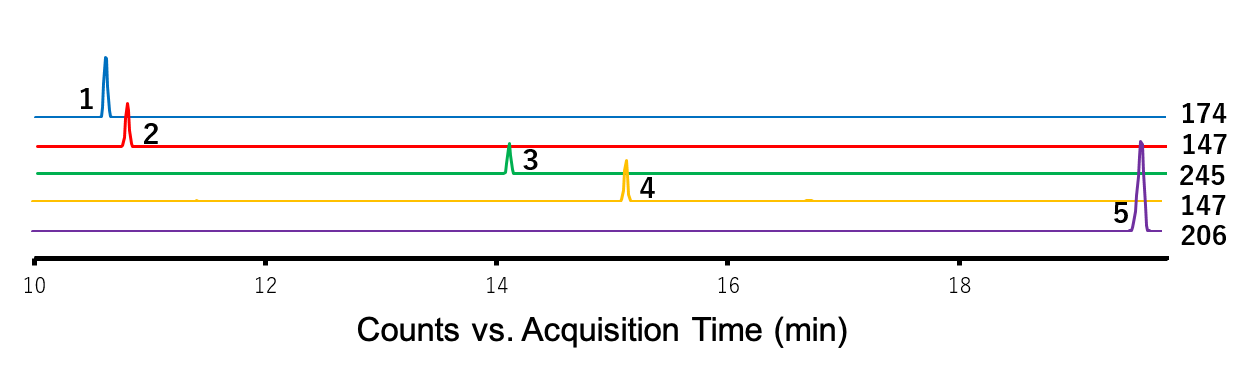

Supplement: SupplementaryFigure1E.png [file KGMR_A_2429408_SM5715.png]

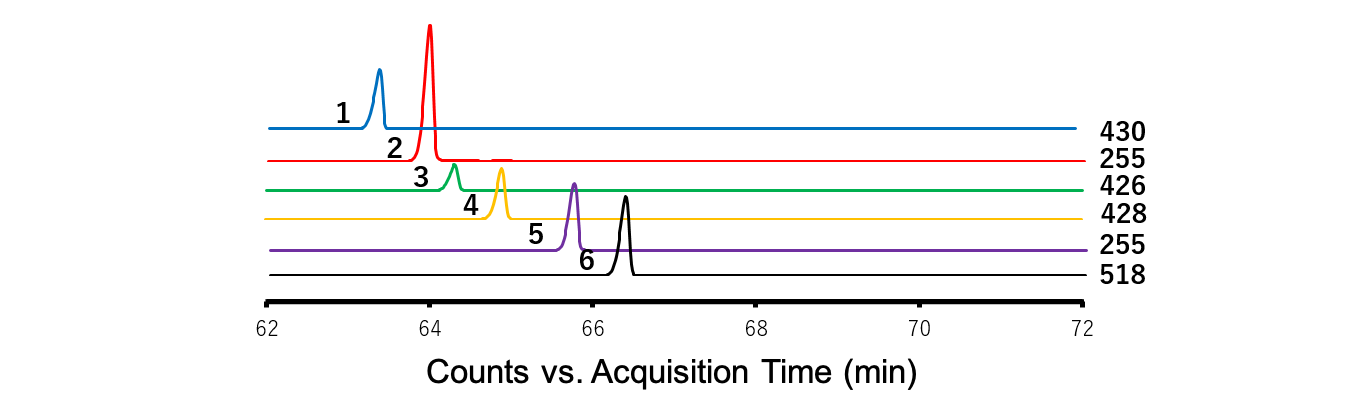

Supplement: SupplementaryFigure1C.png [file KGMR_A_2429408_SM5714.png]

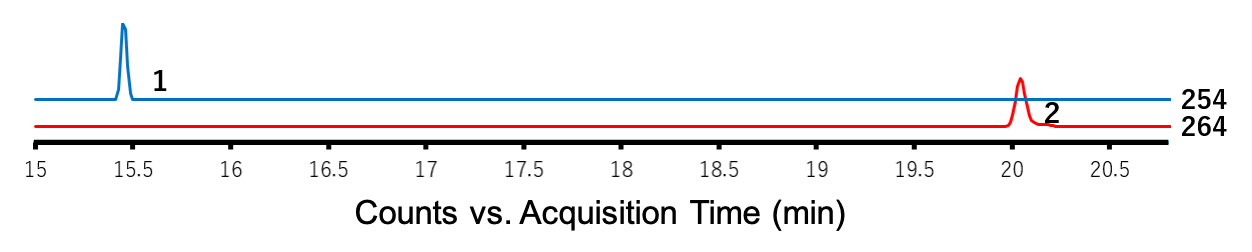

Supplement: SupplementaryFigure1F.png [file KGMR_A_2429408_SM5712.png]

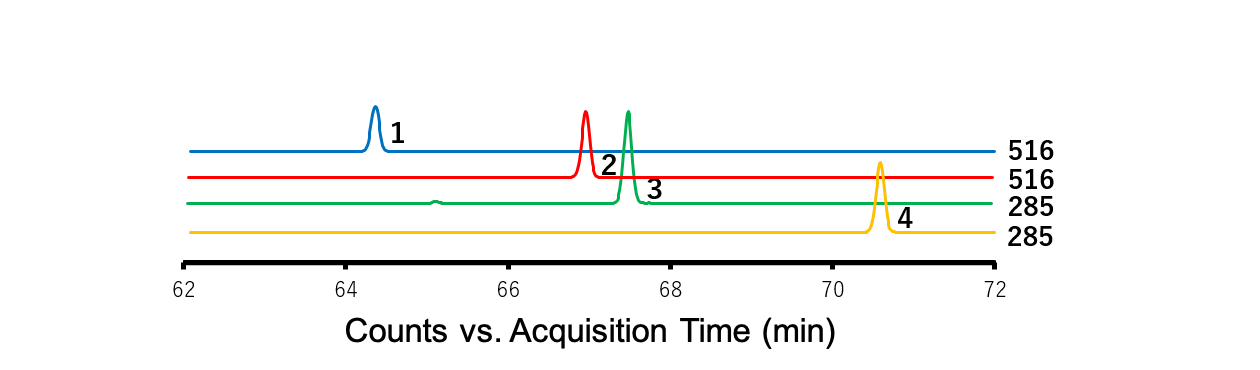

Supplement: SupplementaryFigure1B.png [file KGMR_A_2429408_SM5711.png]
